# Social inhibition as a mediator of neuroticism and depression in the elderly

**DOI:** 10.1186/1471-2318-12-41

**Published:** 2012-08-02

**Authors:** Nahathai Wongpakaran, Tinakon Wongpakaran, Robert van Reekum

**Affiliations:** 1 Department of Psychiatry, Faculty of Medicine, Chiang Mai University, Chiang Mai, Thailand; 2 Department of Psychiatry, University of Toronto, Toronto, Canada; 3 Institute of Medical Sciences, University of Toronto, Toronto, Canada

**Keywords:** Social inhibition, Mediator, Neuroticism, Elderly, Depression

## Abstract

**Background:**

A number of factors, such as demographics, cognitive function, personality and interpersonal relationship) play a role in late-life depression. This study investigates the influence of social inhibition on the inverse emotional stability (neuroticism) and depressive symptoms found in elderly Thai people.

**Methods:**

In total, 123 elderly Thais aged 60 years of age or older were tested using the 64-item Inventory of Interpersonal Problems, Symptom Checklist-90, and the 16 Personality Factors Questionnaire. Hierarchical regression and path analyses were performed in order to identify the relationships among these variables.

**Results:**

The age of the participants ranged from 60 to 93 years old (mean = 71.7; SD = 6.2), and out of the group, 51.2% were male, 56.1% were married and 61.8% were on a low income. The average number of years spent in education among the participants was 7.6 (SD = 5.1). The variables found to be significantly associated with depression were age, intellect, social inhibition and possession of inverse emotional stability (neuroticism). Low levels of emotional stability were most strongly associated with depressive symptoms (standardized regression coefficients −0.29), but this effect was found to be reduced (mediated, to −0.26) by social inhibition. In total, 30% of the total variance could be explained by this model, and there was an excellent statistical fit.

**Conclusions:**

The variables found to be significantly associated with depression were a younger age, as well as lower levels of intellectual skill, social inhibition and inversed emotional stability (neuroticism). It was found that a lack of emotional stability is, along with a younger age, the strongest predictor of depressive symptoms, but can be mediated by social inhibition.

## Background

A number of factors play a role in the appearance of depression in the elderly, these being age, family status, income, cognitive function, personality factors and interpersonal problems. With respect to personality factors, neuroticism is one of the strongest predictors of late-life depression [1-3]. In general, neuroticism manifests itself in the form of negative feelings such as anxiety, a depressed mood, embarrassment and anger. High neuroticism scores indicate emotional instability and a tendency to react to issues, with the associated individuals tending to be emotional, insecure, impulsive, susceptible to psychological distress and vulnerable to stress [4]. Neuroticism can be measured using a variety of instruments; for example, NEO-PI, the 16 Personality Factor (inverse emotional stability) and the Eysenck Personality Questionnaire.

Previous studies have revealed possible mediators between neuroticism and depression; for example, Roelofs et al. [5] found rumination and worrying to be a mediator in depressed individuals, whereas Lee [6,7] found empathy as well as alexithymia to be a mediator. Other mediators that have been studied include daily hassles [8], physical activity [9] and cognitive reactivity [10]; however, these studies did not look at elderly depressed people. Oddone et al. [11] reported that the presence of high levels of neuroticism and low levels of subjective social support may lead to an incomplete recovery in older patients suffering from major depression.

With regard to interpersonal issues, Alden and Bieling [12] found that the ‘social avoidant’ score is positively correlated with depression when measured using the Beck Depression Inventory (BDI) [13]. Pearson et al.’s [14] findings provide further evidence that passive and avoidant coping behaviours are causal factors in depression, as is social inhibition when measured using the Inventory of Interpersonal Problems (IIP). Social inhibition (as with other interpersonal problems) tends to be seen as more stable than a symptom, and requires a greater amount of intervention time in order to affect change [15]. This is also supported by Berghout et al. [16], who studied the changes in symptoms and interpersonal problems found during the first two years of long-term psychoanalytic psychotherapy and psychoanalysis, finding that interpersonal problems change less quickly than the outcome symptoms. Schauenburg et al. [17] found similar results, and commented that according to the items used, IIP problems are more like traits, plus they found that patients with dismissive and introverted styles need a longer time in therapy to affect change when compared to those with styles on the domineering-submissive axis. The authors have come up with three variable types they feel should be the subject of study in terms of relationships; the most enduring being three personality traits, that is, neuroticism (inversed emotional stability), a 'slow to change' state of social inhibition and a 'sensitive to change' state of depression.

With regard to the relationship between introversion, neuroticism and depression, low extraversion (or introversion) have been reported in some studies as having a negative correlation with depression [18-24]. However, the role of introversion as a risk factor for depression is less visible, or at least less pronounced, than that of neuroticism [25,26]. Denollet [27,28] developed the Type D scale 16 (DS16) based on a two-construct personality which includes (i) negative affectivity (NA), which denotes a stable tendency to experience negative emotions. This trait has also been conceptualized as neuroticism [29,30] - with the NA sub-scale correlated with neuroticism in the NEO-FFI (r = 0.68) and the Eysenck Personality Questionnaire (r = 0.64) [31], and (ii) social inhibition (SI), denoting the stable tendency to inhibit expressions of emotion during social interaction. SI negatively correlates with the extraversion scale from the NEO-FFI (r = − 0.52), and the Eysenck Personality Questionnaire (r = − 0.65) [31]. Even though most of Denollet’s work was carried out in patients with a medical illness present, such as coronary heart disease, it provides evidence that NA and SI have an impact on depression [32]. A type D personality, as described by Denollet, refers to an individual with high NA and high SI levels, and after analysis of its predicting ability, has been categorized into a Type D and non-type D grouping by using a median split cut-off. As well as considering the moderating effects of these two separate variables, it would be interesting to see whether social inhibition has a mediating effect on negative affectivity, because at least two studies have found this to be the case. For example, Denollet et al. support the hypothesis that the effects of social inhibition plus negative emotions, rather than negative emotions *per se*, can predict depression [33], whereas Uliaszek et al. discovered that neuroticism plays a role in the relationship between depression and interpersonal stress (r = 0.32), though their evidence failed to support a role for neuroticism in the association between depression and non-interpersonal stress (r =0.08)[34]. This implies that interpersonal issues can act as mediators of neuroticism.

The present study, therefore, an aims to explore the mediating effect social inhibition has on neuroticism, as this has not been reported in elderly people with depression before. It is hoped that a greater understanding of this relationship will ultimately help to improve preventive and therapeutic interventions among depression patients.

## Methods

This work represents the secondary analysis of data taken from a previous study [35] carried out in 2009, and which was approved by an independent ethics committee at the Faculty of Medicine, Chiang Mai University.

### Participants

The authors analyzed the data taken from a national survey held in 2009 into the interpersonal problems found among Thai people [35]. Five sites from five different regions of Thailand were selected (with each site taken from a province representing a given region). These sites were not randomly selected but were chosen specifically because they were already part of an established research network. The total N, and the n per site, were calculated by a statistician according to the population size in each region. Participants were selected using convenience sampling after announcements were issued in the study communities. In total, 194 people aged 60 years and over were invited, and 126 participated, though the data for three people were subsequently excluded due to the fact that they were incomplete, leaving 123 participants to be included in the analysis.

Participants provided demographic data, plus responded to the Thai version of the IIP, the Sixteen Personality Factor (16 PF) and Symptom Checklist (SCL)-90 questionnaires.

### Instruments

#### Thai version of the 64-item inventory of interpersonal problems (IIP-64)

IIP-64 [36] is a self-report questionnaire which measures interpersonal difficulties across eight sub-scales, those derived from the following dimensions: affiliation (from hostile/cold to friendly behaviour), domineering (from submissive to controlling behaviour; for example, “I try to control other people too much”), vindictive (for example, “I am too suspicious of other people”), cold (for example “I keep other people at a distance too much”), non-assertive (for example, “I find it difficult to let other people know what I want”), socially inhibited (for example, “I am too afraid of other people”), overly-accommodating (for example, “I let other people take advantage of me too much”), self-sacrificing (for example “I put other people’s needs before my own too much”) and intrusive/needy (for example “I find it difficult to spend time alone”). The Thai version of IIP-64 demonstrates a good overall internal consistency [35] of α = 0.95, then for domineering α = 0.79, vindictive α = 0.75, cold α = 0.82, socially inhibited α = 0.79, non-assertive α = 0.78, overly-accommodating α = 0.74, self-sacrificing α = 0.75, and for intrusive-needy α = 0.75 [35].

#### Depression dimension of the symptom checklist-90 (SCL-90)

The SCL-90 [37] is a self-report questionnaire composed of 90 items, and is used to assess psychological problems and symptom distress. Each item assesses symptom severity on a five-point scale, from 0 (not at all) to 4 (extremely). It has three global scores: the General Symptom Index (GSI), the Positive Symptom Total (PST) index and the Positive Symptom Distress Index (PSDI). The measure reports on nine symptom characteristics, these being: somatization, obsessive-compulsive, interpersonal sensitivity, hostility, depression, anxiety, paranoid ideation, phobic anxiety and psychoticism. The measure was developed for use with people aged 15 to 67 years, and the Thai version used in this study was developed by Chooprayoon L. [38], having demonstrated good internal consistency and validity (it is a known group technique). For the present study, Cronbach’s alpha was found to be 0.92 [39].

#### Thai version of the 16 personality factors questionnaire (16 PF)

The 16 PF was developed by Cattell [40], and is a personality measurement tool which categorizes personality based on sixteen characteristics, these being: warmth, intellect, emotional stability, dominance, liveliness, rule-consciousness, social boldness, sensitivity, vigilance, abstractedness, privateness, apprehensiveness, openness to change, self-reliance, perfectionism and tension, each of which reflect an individual’s adjustment, problem-solving and event perception styles. The 16 PF contains 187 items with three choices available for each item. Scoring can be rated by giving a score of 1 or 2, or by comparing against a standardized score, and interpretation is given as graphical sten scores. The 16 PF has been shown to have adequate validity and a high test-retest reliability: 0.80 (0.69-0.87) over a two-week interval, and 0.70 (0.56-0.79) over a two-month interval [41]. The Thai version has also been shown to have a good internal consistency (0.61- 0.88) [42].

### Statistical analysis

In this study, distributions and descriptive statistics were examined for all the variables, with outlying data points reduced to three SD above the mean in order to reduce their influence on the analysis. Pearson’s correlation coefficients were used to examine the links between depression and the four variables that might confound any link between inversed emotional stability and depressive symptoms, these being: age, education, household income and intellect. Intellect is not regarded as a personality factor but rather as a function of intellectual ability; therefore, it was treated as a variable to be controlled [43,44].

According to Baron and Kenny [45], in order for social inhibition to be a mediator, it must be correlated with both the predictor (inversed emotional stability) and the outcome (depressive symptoms), and in addition, the predictor must be linked with the outcome. If these criteria are met, hierarchical linear regression analyses can be used to test whether social inhibition has a mediator effect, that is, whether it reduces the regression coefficient - the link between inversed emotional stability and depression. In this study, age, education, household income, and intellect were all controlled.

In order to test for model fitness, AMOS 18 was used to conduct path analysis via a maximum-likelihood estimation method; with all single indicators allowed to be correlated. Two fit indices commonly used in the CFA literature were used to evaluate the model fit, these being the goodness-of-fit index (GFI) [46] and the comparative fit index (CFI) [47]. The root mean square error of approximation (RMSEA) is an evaluation statistic that is relatively unaffected by sample size, and is thus suitable for assessing models of differing complexity [47,48]. Hierarchical regression analysis was used as well as path analysis to depict the direct and indirect effects of the involved factors.

## Results

The age of the participants ranged from 60 to 93 years old (mean = 71.7; SD = 6.2), and out of the group, 51.2% were male, 56.1% were married and 61.8% were on a low income. The average number of years spent in education among the participants was 7.6 (SD = 5.1) (see Table1). No significant link was found between gender and depression scores (*t = −1.00, df = 121*, *p = 0.32*), so gender was not included in the regression models. Age and intellect, but not income, were found to have an influence on depression; the higher the age, the less intellect is used and the more depressed a person is likely to be ( *R*^*2*^ = 0.243, p < 0.01; *R*^*2*^ = 0.212, p < 0.01, respectively) (see Table2).

**Table 1:** Descriptive statistics

**Descriptive Statistics**	**Mean**	**SD.**
Age	71.72	6.72
Education	7.61	5.16
Income	2.02	1.71
Intellect	4.47	1.83
Social inhibition	16.11	6.12
Inversed emotional stability	4.28	1.49
Depressive symptoms	0.71	0.57
Intrusive-needy	14.44	5.58

**Table 2:** Inter-correlation matrix

	**1**	**2**	**3**	**4**	**5**	**6**	**7**
Age	1	−0.358	−0.305	−0.09	0.114	0.004	0.326
Education	−0.358	1	0.75	0.217	−0.194	−0.073	−0.286
Income	−0.305	0.75	1	0.277	−0.105	−0.098	−0.265
Intellect	−0.09	0.217	0.277	1	−0.097	0.036	−0.29
Social inhibition	0.114	−0.194	−0.105	−0.097	1	−0.178	0.304
Inversed emotional stability	0.004	−0.073	−0.098	0.036	−0.178	1	−0.281
Depressive symptoms	0.326	−0.358	−0.305	−0.09	0.114	0.004	1

A series of hierarchical regression analyses were conducted to (1) evaluate the contribution of inversed emotional stability as a predictor of depressive symptoms, and (2) to evaluate the contribution of the submissive interpersonal style of social inhibition towards mediation of the effects of inversed emotional stability on prospective depressive symptoms, adhering to the process used for testing mediation as outlined by Baron and Kenny [45]. SPSS diagnostics were examined to ensure that the hierarchical regression models were not biased due to multi-collinearity, or due to the influence of outliers and residuals. Due to one outlier with a standardized residual of > 0.3, the depression score was log transformed. The transformed data indicated that for all VIF scores of < 10 and all tolerance statistics of > 0.2, there were no standardized residuals with an absolute value of > 0.3, and so the assumption regarding independent errors was met.

**Figure 1:**
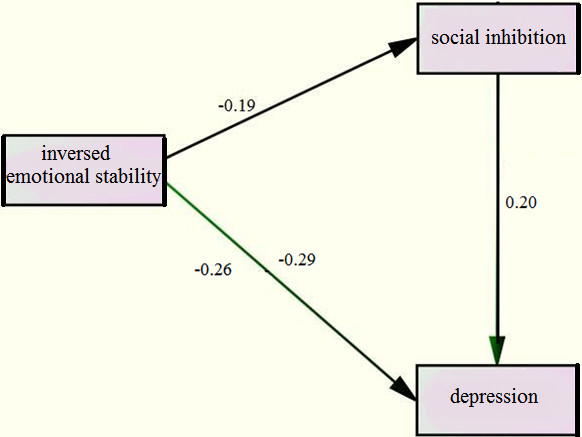
Model showing the mediating role of social inhibition in the relationship between inversed emotional stability and depression (below the regression line).

Links between potentially confounding variables and depressive symptoms were also examined. Table3 presents the results of two hierarchical regression models used to test whether social inhibition mediates the link between inversed emotional stability and depressive symptoms. Age, years in education, household income and intellect were included in step 1 as covariates, and accounted for 19% of the variance in depression. Inversed emotional stability was introduced in step 2 and was a significant predictor of depression scores, even after controlling for the covariates in step 1 - explaining an additional 8% variance. Social inhibition was introduced in step 3, explaining an additional 3% of the variance and reducing the regression coefficient for inversed emotional stability from beta −0.29 to −0.26, supporting the hypothesis that social inhibition mediates between neuroticism and depressive symptoms (see Figure 1). Nevertheless, the effect sizes for the main link in this mediational model were moderate (d = 0.37) [49].

**Table 3:** Hierarchical regression analysis scores for those variables predicting depression (n = 123)

**Variable**		**B**	**SE B**	**β**	**ΔR**^**2**^
Step 1	Age	0.016	0.006	0.252**	0.192
	Education	−0.010	0.011	−0.121	
	Income	−0.008	0.033	−0.033	
	Intellect	−0.055	0.021	−0.232**	
Step 2	Age	0.016	0.006	0.243**	0.083
	Education	−0.010	0.010	−0.123	
	Income	−0.017	0.031	−0.067	
	Intellect	−0.051	0.020	−0.212**	
	Inversed emotional stability	−0.085	0.023	−0.290**	
Step 3	Age	0.015	0.005	0.234**	0.033
	Education	−0.007	0.010	−0.077	
	Income	−0.022	0.031	−0.085	
	Intellect	−0.048	0.019	−0.201*	
	Inversed emotional stability	−0.075	0.023	−0.255**	
	Social inhibition	0.013	0.006	0.189*	

*p < 0.05; ** *p < 0*.01.

Finally, it was found that the regression model contributed a significant amount of variance to depressive symptoms (30%).

A specification search was adopted in order to find the model with the best fit, and the final model used displayed an excellent fit: *χ*^2^ = 4.23, df = 6, p = 0.65, CFI = 1.00, TLI =1.07, NFI = 0.95, GFI = 0.99, RMSEA = 0.00 and SRMR = 0.03, with all paths significant (p < 0.05).

## Discussion

Similar to previous reports [1-3,14,50-52], in this study personality factors were found to predict depressive symptoms. The authors also found that social inhibition plays a role as a mediator between inversed emotional stability and depressive symptoms. or put another way, when mediated by social inhibition, the less emotional stability that exists the more severe the depression is likely to be. These results indicate that social inhibition has a relationship with both personality factors and depression, to a medium level of significance.

It is important to note that this is not a full mediating effect, meaning that those who have the neuroticism trait will be prone to develop depression when they are socially inhibited, but that there are still other mediators involved.

What can be discerned with regard to the mediating effect of social inhibition on both relationships? The authors hypothesize that there may be some possible explanations for these results. First, our results support Uliaszek et al.’s idea that social inhibition may affect the relationship between neuroticism and depression by tapping into interpersonal stress. This corresponds to previous research carried out by Kendler et al., who found that neuroticism tends to more strongly predict interpersonal than non-interpersonal life stress [53]. This shows how important interpersonal issues are in the relationship between neuroticism and depression, in that depression may occur as a chronic negative effect of neuroticism through the interpersonal process; and may even worsen when a person with neuroticism is socially inhibited. Second, social inhibition is found to be related to self-criticism, which is linked with depression, as examined by Alden & Bieling [12], Clara et al. [54] and Dunkley et al. [55]. Interestingly, Cox et al. [56] found that avoidant personality disorder is also related to self-criticism. In addition, social inhibition is related to avoidant personality disorder, which has been found to be linked to depression [57,58]. Lastly, social inhibition may lead to loneliness, which will worsen when combined with a high level of neuroticism [2].

Taken as a whole, social inhibition would appear to be another risk factor for depression, as evidenced by previous studies. What the authors are proposing here is that it is not only another risk factor, but also a mediator for neuroticism, or to put it another way, it minimizes the importance of neuroticism *per se* in the development of depression.

According to the other variables taken into account (age, income, education and intellect – those controlled), the authors observed that only personality factors are significant predictors of depression, and this finding supports earlier research [59,60] which shows that inversed emotional stability is one of the strongest predictors of depression in the elderly.

In addition, cognitive ability, as reflected by intellect in the 16 PF, correlated with age and depression, a finding also supported by previous studies [61,62]. Although income is deemed to be important, in comparison to other factors it contributes the least to depression. This finding might be attributed to the fact that most of the data from this study were collected from elderly people in the low income bracket. Moreover, in a collectivistic type of society as found in Thailand - where the elderly mostly stay with their children, elderly people are financially supported and do not have to acquire much wealth to live comfortably into old age. Psychological well-being, psychological support and recognition from the younger population are considered to be major requirements for elderly Thais [63], and the percentage of total variance explained in this regard by our model was 30%. However, with questions unanswered in this area, more research and discussion is needed on this topic, in order to examine the other variables that have an important bearing on depression in the elderly.

Even though depressive symptoms tend to occur in those suffering from emotional insecurity, the problem of social inhibition may be another aspect to look for and to study, because, as our results show, the lower the level of social inhibition the less likely individuals are to be affected by neuroticism, or to develop depressive symptoms. Given the fact that depression is related to an avoidant personality, self-criticism, loneliness, hostility and submissive behaviours, all of which share inversed emotional stability and social inhibition (hostile-submissive), to move patients from the hostile to the friendly pole, even though it is not as easy as appears, has been proven to be a better outcome despite the fact that the personality problems still exist [64-66]. This might be a reminder for clinicians that, when dealing with depressed patients with a high score on neuroticism, they should be aware of social inhibition problems and explore associated causes. Even though social inhibition and depression can affect each other, social inhibition tends to exist for longer and it may require a search for its underlying causes in order to prevent the recurrence of depression. Clinicians might thus focus on interpersonal behaviours related to social inhibition, because even though they may not change as quickly as depressive symptoms, they are likely to change faster than personality traits such as neuroticism.

### Limitations

The study group consisted of a non-clinically diagnosed sample of depressed Thai people, which limits the inference of the study findings to clinically depressed Thais. Furthermore, this study used a cross-sectional structure; therefore, it is not possible to make cause-effect determinations based solely on the data here, so future longitudinal or experimental studies are needed to facilitate an evaluation of causality. Finally, the data in this study were collected using self-reporting scales, so the use of other evaluation tools such as family member and caregivers’ reports, clinician-rated diagnoses, and peer or family assessments of interpersonal and personality dimensions, may decrease the ‘subjectivity’ limitation.

## Conclusion

Age and personality factors in relation to inversed emotional stability (neuroticism) and the interpersonal problem of social inhibition, all play a strong role in predicting depression in the elderly. Further studies are needed; however, using a larger sample of clinically depressed elderly people, and with a longitudinal follow-up, in order to study how social inhibition really impacts upon depression.

## Competing interests

The authors declared that they have no competing interest.

## Authors’ contributions

NW and TW conceived of and designed the research, supervised the data collection and wrote the manuscript, while RvR assisted with the research design and the writing of the manuscript. TW was responsible for the statistical analysis. All authors have read and approved the final version of this manuscript.

## Pre-publication history

The pre-publication history for this paper can be accessed here:

http://www.biomedcentral.com/1471-2318/12/41/prepub
